# Longitudinal assessment of the temporal stability and predictive validity of the Revised Paranormal Belief Scale

**DOI:** 10.3389/fpsyg.2022.1094701

**Published:** 2023-01-27

**Authors:** Neil Dagnall, Andrew Denovan, Kenneth Graham Drinkwater

**Affiliations:** ^1^Department of Psychology, Manchester Metropolitan University, Manchester, United Kingdom; ^2^Department of Psychology, University of Huddersfield, Huddersfield, United Kingdom

**Keywords:** longitudinal assessment, predictive validity, Revised Paranormal Belief Scale, test–retest reliability, health outcomes

## Abstract

The Revised Paranormal Belief Scale (RPBS) is the prevailing measure of supernatural credence. However, there exists only limited evidence to support the temporal stability and predictive validity of the instrument over time. Acknowledging this, the present study assessed the test–retest reliability of the RPBS using a large, heterogeneous sample across multiple trials. In addition, predictive validity was tested using a longitudinal statistical model, which focused on allied health outcomes (Perceived Stress and Somatic Complaints). A sample of 1,665 (*M*_age_ = 54.40, 853 females, 804 males, five non-binary and three not disclosing of gender) completed study measures at three time points separated by 2 month intervals. Prior to assessing temporal stability, assessment of structural validity and longitudinal invariance occurred. Test–retest reliability of the RPBS was in the moderate to high range across time intervals, and good internal consistency was observed. Furthermore, satisfactory stability coefficients existed for RPBS subfactors. Data-model fit for the predictive model was acceptable. Belief in the paranormal explained low variance over time in Perceived Stress and Somatic Complaints (between 2.4 and 4.2%). Findings supported the stability and reliability of the RPBS. In addition, they aligned with the notion that paranormal belief in the absence of high scores on cognitive-perceptual factors (e.g. transliminality and schizotypy), has a benign influence on perceived health.

## Introduction

The Revised Paranormal Belief Scale (RPBS) (Tobacyk, 2004) is the most prevalently employed measure of supernatural credence (Drinkwater et al., 2017). The scale is a modified version of the Paranormal Belief Scale (PBS) (Tobacyk and Milford, 1983), which was created in response to intensified scholarly interest in paranormal phenomena. Central themes guiding development were novel forms of communication; existence of a founding universal principle; the notion of existence as comprising body, mind/spirit/soul and afterlife and the conceptualisation of reality as perceived rather than veridical (Rominger et al., 2022).

The synergy of phenomena encompassed by these themes encapsulated a breadth of beliefs, which were categorised as ‘religion, psi (clairvoyance, precognition, telepathy and psychokinesis), the occult, witchcraft, superstitions, the supernatural and extraordinary and extra-terrestrial life forms” (Tobacyk and Milford, 1983, p. 1025). Although other belief measures were in use at the time, these were constrained by assumptions about the nature of paranormal belief (Irwin, 2009; Dagnall et al., 2010a,b). Explicitly, content reflected preconceived views on dimensionality (unidimensional, Randall and Desrosiers, 1980 vs. multidimensional, Scheidt, 1973) rather than domain content. Noting this limitation, as part of the PBS development Tobacyk and Milford (1983) identified the structure of paranormal belief.

The PBS originated from analysis of 61 statements. These comprised items from existing instruments and newly constructed questions. Item selection was determined by criteria derived from current scientific understanding and orthodox notions of reality (Broad, 1953; Braude, 1978). The item pool was administered to psychology students (*N* = 391) at Louisiana Tech University. Analysis of scores identified seven orthogonal (uncorrelated) factors (i.e. Traditional Religious Beliefs, Psi, Witchcraft, Superstition, Spiritualism, Extraordinary Lifeforms and Precognition). Some researchers refer to these as the Big Orthogonal Seven model (BOS). Within the PBS, emergent factors were conceptually congruent; items reflected domain content and were internally consistent. The extracted 25-item scale employed a five-point Likert scale (1 = strongly disagree to 5 = strongly agree) and demonstrated psychometric integrity (convergent and discriminant validity). The BOS was important because it informed the content and structure of the subsequent revision, the 26-item RPBS (Tobacyk, 2004).

Modifications included enhancement of the Precognition subscale to ensure that items accurately evaluated construct domain, and amendments to the Extraordinary Lifeforms and Witchcraft subscales. To reduce range restriction, a seven-point Likert scale was adopted. These refinements improved subscale validity, reliability and Western cross-cultural standardisation. The importance of the RPBS is demonstrated by the fact that researchers later translated the scale into several languages (e.g. French, Bouvet et al., 2014; Spanish, Díaz-Vilela and Álvarez-González, 2004; Latvian, Utinans et al., 2015, Urdu, Rao et al., 2021 and Chinese, Shiah et al., 2010).

Investigators use the RPBS for several reasons. Firstly, the RPBS is an established instrument with attested psychometric properties. Secondly, the scale has featured prominently in published research (Dagnall et al., 2010b; Drinkwater, 2017). This signifies that it is a recognised, established measure of paranormal belief. Thirdly, since researchers have used the RPBS extensively it is possible to draw meaningful comparisons across studies. Fourthly, the RPBS in comparison to the Australian Sheep-Goat Scale (ASGS) (Thalbourne and Delin, 1993) assesses a wider range of construct content. The ASGS is another commonly used measure of paranormal belief. Social scientists tend to use the RPBS because of its breadth, whereas parapsychologists employ the ASGS because it focuses on core discipline domains (i.e. extra-sensory perception, psychokinesis and life after death) (Drinkwater et al., 2018a). Moreover, while the ASGS had been defined as a multidimensional instrument, Rasch analysis correcting for items displaying differential item functioning, recommends a single New Age Belief solution (Lange and Thalbourne, 2002) comprising only extra-sensory perception and psychokinesis.

Finally, the presence of subscales within the RPBS allows researchers to assess specific beliefs and/or examine whether belief type interacts differentially with psychological factors. Illustratively, the superstition subscale has featured as a standalone measure within published articles (see Wiseman and Watt, 2004; Dagnall et al., 2007, 2009). With reference to identifying psychological variations as a function of belief type, several studies report nuanced differences that were obscured by overall effects (e.g. Irwin et al., 2012a,b; Williams and Roberts, 2016; Kumar et al., 2020).

A caveat with the use of subscales is that alternative factorial solutions exist. Typical models are the original seven factors, a five-factor structure (Traditional Religious Belief, Psychic Beliefs, Superstition, Witchcraft and Anomalous Natural Phenomena) (Lawrence, 1995) and a two-factor purified model (New Age Philosophy, NAP and Traditional Paranormal Beliefs, TPB) (Lange et al., 2000). The latter is statistically rather than conceptually driven. Hence, emergent clusters combine subscale items and are defined in terms of function (i.e. influence over external events). NAP provides control at the individual/personal level, and TPB over social cultural factors. Despite correcting for differential item functioning, relatively few papers have used the two-factor in preference to the original RPBS solution (Drinkwater et al., 2018b). It is for this reason that the present paper focused on the stability of the seven factors and overall score over time. These solutions are the most frequently reported indices of paranormal belief (particularly the global score).

Notwithstanding debates about the factorial structure of the RBPS, there is currently only limited evidence to support the measure’s temporal stability. This issue is not specific to the RPBS as reliability across time, context and user are generally poorly reported within psychological literature (Aldridge et al., 2017). In this context, assessment of test–retest reliability is vitally important to establishing the psychometric integrity of the RPBS and determining the long-term effects of paranormal belief on health outcomes. Test–retest reliability is the systematic assessment of consistency, reproducibility and agreement between two or more respondent scores on the same measurement instrument under equivalent conditions. To demonstrate accuracy respondent scores must be similar across tests. This demonstrates that variations represent changes in the individual and are not the consequence of measurement inconsistency.

Test–retest reliability is also important because it complements validity by evidencing that a scale produces stable measurements. Hence, test–retest reliability is particularly important when evaluating the efficacy of treatments and interventions. In the current study, RPBS temporal stability was vital to determining the degree to which paranormal belief accurately predicted health-related outcomes over time.

A vital consideration when establishing test–retest is the gap between scale completions. The interval needs to be sufficient to evidence adequate stability, but not too long to result in significant attrition or opportunities for extrinsic factors to influence scores. Hence, with health measures intervals of 1–2 weeks are typically used (Polit, 2014). This is often constrained by practicality and hence, test–retest within validation papers often represents intervals of convenience (Watson, 2004).

In the case of the PBS, Tobacyk and Milford (1983) assessed the test–retest reliability of the PBS over a 4-week interval with a 25-subject sample and reported subscales correlations between 0.60 and 0.84. RPBS modification improved these figures (Tobacyk, 2004). Using a sample of 40 university students, 4-week test–retest reliabilities were Precognition 0.81, Witchcraft 0.93, Extraordinary Lifeforms 0.91, Traditional Religious Belief 0.95, Psi 0.71, Superstition 0.89 and Spiritualism 0.91. The overall scale test–retest was 0.92. While these figures indicate good to excellent test–retest reliability (Cicchetti, 1994; Fleiss, 2011), samples were restricted to students and the gap between testing points was only 4-weeks.

While this approach is typical within psychology, to effectively establish test–retest reliability, researchers need to consider the nature of the construct under observation and the extent to which they wish to extrapolate findings. This is vital since some psychological phenomena (e.g. mood and affect) fluctuate as a function of internal and external factors, whereas others (e.g. personality) remain relatively stable. Hence, paranormal belief is influenced by a combination of situational and dispositional factors. Additionally, belief types differ in lability. For example, traditional religious beliefs, which include notions of heaven and hell, are typically inculcated and enduring. While superstition varies in accordance with external factors that create negative affect (Padgett and Jorgenson, 1982; Dagnall et al., 2007). This aligns with the notion that superstitious belief and magical thinking serve as mechanisms for coping with environmental stress and uncertainty (Malinowski, 1948; Jahoda, 1969; Frost et al., 1993). Hence, these beliefs increase during periods of life pressure and unpredictability.

Noting these factors and the restricted nature of validation studies, it was necessary to further assess the test–retest reliability of the RBPS using a large, heterogeneous sample, across multiple time points over an extended period. This allowed the authors to thoroughly examine scale stability and provide further insights into the nature of belief. Explicitly, determine whether supernatural credence remained relatively unchanged over time or fluctuated in accordance with internal and external factors.

### Paranormal belief and well-being

Recent research examining relationships between paranormal belief and well-being indicates that supernatural credence is benign in the absence of productive and disorganised cognitive-perceptual and psychopathological factors (Drinkwater et al., 2021; Dagnall et al., 2022a). It is the interaction with/and indirect influence of these factors, which explain negative belief-related outcomes. In the case of interactions, higher levels of belief combine with correlated constructs (i.e. schizotypy, transliminality and reality testing deficits) to create psychological profiles, which are characterised by a steady flow of unstructured mentation, and an overreliance on internal, intra-psychic activity and subjective interpretation. Psychological profiles with these characteristics are most strongly associated with lower well-being and poorer psychological adjustment.

In this context, believers comprise a set of subgroups and the effects of paranormal belief are defined by scores on concomitant cognitive-perceptual and psychopathological factors (Dagnall et al., 2011; Denovan et al., 2018). Hence, subgroups with higher psychopathology scores (schizotypy, depression and manic-depressive experience) tend to report lower well-being and poorer psychological adjustment. Subsequent work confirmed and extended these findings. Specifically, *via* a longitudinal design, Dagnall et al. (2022c) observed that over time the highest scoring psychopathology profile (vs. lower) was associated with higher negative and lower positive well-being.

With reference to indirect effects, within paranormal believers, transliminality and specific psychopathology-related variables in combination (i.e. the Unusual Experiences and Cognitive Disorganisation subscales of schizotypy and manic-depressive experience) predicted vulnerability to negative well-being outcomes (see Dagnall et al., 2022d). Transliminality is important because elevated levels indicate hypersensitivity to psychological material (both internal and external; Thalbourne, 1998). This suggests a reduction in the ability to attenuate and limit the stream of cognitive-perceptual information. It also potentially explains why transliminality is a prognosticator of psychopathology. Unusual Experiences reflect positive schizotypy (i.e. magical thinking, perceptual aberrations and hallucinations), and Cognitive Disorganisation thought disorder and additional allied aspects of psychosis (i.e. poor attention, decision-making and social anxiety). Manic-depressive experience is also important because higher scores reflect issues with affect regulation.

Correspondingly, using network analysis Dagnall et al. (2022b) observed that transliminality connected paranormal belief, positive schizotypy and psychopathology. Additionally, the association between transliminality and well-being was bridged by depressive experience. These findings indicate that transliminality embodies features such as lower cognitive flexibility (Peters et al., 1999) and heightened sensory information flow (i.e. reduced latent inhibition and hypersensitivity) (Carson, 2011), which are present in more extreme forms within psychosis (Waltz, 2017; Calvo et al., 2021). These shared features likely explain why transliminality correlates positively with supernatural credence and signifies vulnerability to psychopathology.

### The present study

Acknowledging the limited work assessing the temporal stability of the RPBS, the present paper examined the test–retest reliability of the instrument with a large, heterogeneous sample across multiple time points. It was anticipated that this would establish temporal consistency and indicate whether the RPBS was similarly predictive of perceived stress (Cohen and Williamson, 1988) and somatic complaints (Gierk et al., 2014) at different intervals. These allied health outcomes were selected because previous research has reported relationships between paranormal belief and perceived stress (Lasikiewicz, 2016) and somatic complaints (Dagnall et al., 2022d; Laythe et al., 2022), and researchers generally acknowledge that stress and related psychosomatic symptoms are negatively associated with psychological and physical health (Wei et al., 2020).

Although causes of somatic symptoms are not currently well understood (Henningsen et al., 2007), researchers report that stress generally contributes to perceptions and interpretations of somatic symptoms (Witthöft and Hiller, 2010; Li et al., 2016). A model that potentially explains the relationship between stress and somatic symptoms is the stress-system model for functional somatic symptoms (Kozlowska, 2013). This views stress as a brain–body interaction underpinned by multiple interconnected systems. These include the hypothalamic–pituitary–adrenal axis, autonomic nervous system, immune-inflammatory system and brain stress systems allied to emotional states, salience detection and pain. Collectively, these protect individuals from threats. Since these function in a holistic, integrated manner activation of any system can initiate or dysregulate other systems. Accordingly, inappropriate or sustained activation of the stress system can affect its responsiveness and sensitivity, resulting in somatic symptoms (i.e. excessive awareness of physical sensations).

This manifests as persistent unspecific symptoms that cause individual concern and facilitate medical consultation, but are not classified as disease (Roenneberg et al., 2019). Moreover, stress-related somatic symptoms adversely affect emotional distress, which reciprocally *via* negative cognitive bias can increase reporting of somatic concerns (Wei et al., 2020). This model aligns with studies that report positive correlations between perceived stress and somatic complaints (e.g. Roenneberg et al., 2019). A further reason for selecting perceived stress and somatic complaints is that they are assessed over relatively short periods (the past month and 7-days respectively). Hence, scores are subject to variations in accordance with life pressures.

Commensurate with previous research, it was hypothesised that paranormal belief would remain stable over time and that supernatural natural credence, in the absence of other psychological factors (i.e. transliminality), would explain only low levels of variance in perceived stress and somatic complaints.

## Materials and methods

### Sample

This study used a longitudinal approach. Respondents (*N* = 1,665) completed study measures at three time points separated by two-month intervals. Sample mean age (*M*_age_) was 54.40, range = 18–91, and gender was balanced, 853 females (*M*_age_ = 55.49, range = 18–91) and 804 males (*M*_age_ = 53.34, range = 18–86). Five participants were non-binary (*M*_age_ = 41.80, range = 23–69), and three preferred to not disclose gender (*M*_age_ = 48.66, range = 33–63). To participate, respondents had to be at least 18 years of age and free from a diagnosed mental illness.

Recruitment of participants occurred *via* Bilendi in 2020/2021, who source responses from a pool of individuals with an interest in participating in research studies. A large and representative sample was requested alongside a minimum age (18 years). Kees et al. (2017) report that online panel data are analogous to data collected *via* traditional means.

### Measures

#### Revised Paranormal Belief Scale

The Revised Paranormal Belief Scale (RPBS; Tobacyk, 2004) assessed belief in the supernatural. The measure comprises 26-items, which are presented as statements (e.g. ‘Black Magic really exists’). Participants indicate their level of endorsement on a Likert scale (ranging from 1 = Strongly Disagree to 7 = Strongly Agree). Total scores range from 26 to 182 with higher scores representing greater paranormal belief. The RPBS comprises seven subscales: Traditional Religious Belief (4-items; life after death and heaven and hell), Psi (4-items; psychic powers), Witchcraft (4-items; casting skill and magical powers), Superstition (3-items; bad luck), Spiritualism (4-items; non corporeality), Extraordinary Lifeforms (3-items; existence of yet to be established entities) and Precognition (4-items, predicting future events). The RPBS overall and at the subscale level was internally reliable (see Table 1).

**Table 1:** Descriptives and reliability for RPBS, PSS, PSS factors, SSS-8 and RPBS factors.

Variable	*M*	*SD*	Skew	Kurt.	*α*
PB T1	35.19	19.44	0.25	−0.81	0.94
PSS T1	16.29	7.82	0.11	−0.27	0.87
PSS Distress T1	9.12	6.05	0.26	−0.67	0.92
PSS Coping T1	7.17	3.59	0.38	−0.01	0.84
SC T1	16.28	7.08	0.85	0.04	0.87
PB T2	35.39	19.92	0.22	−0.91	0.94
PSS T2	16.27	7.79	0.07	−0.27	0.87
PSS Distress T2	9.01	5.98	0.24	−0.71	0.92
PSS Coping T2	7.26	3.69	0.32	−0.07	0.85
SC T2	16.35	7.07	0.84	0.02	0.87
PB T3	34.96	20.35	0.24	−0.97	0.95
PSS T3	16.16	7.96	0.12	−0.21	0.87
PSS Distress T3	8.80	6.13	0.36	−0.59	0.92
PSS Coping T3	7.36	3.84	0.31	−0.21	0.86
SC T3	16.24	7.39	0.89	0.05	0.89
**RPBS factors**					
TRB T1	4.69	3.82	0.30	−1.11	0.79
TRB T2	4.82	3.87	0.25	−1.17	0.80
TRB T3	4.90	3.92	0.20	−1.22	0.82
PSI T1	5.32	3.95	0.08	−1.27	0.85
PSI T2	5.39	4.04	0.05	−1.31	0.87
PSI T3	5.25	4.09	0.09	−1.36	0.88
WITCH T1	5.10	3.78	0.10	−1.15	0.82
WITCH T2	5.11	3.84	0.09	−1.21	0.84
WITCH T3	5.03	3.90	0.15	−1.22	0.85
SUP T1	2.80	3.00	0.65	−0.91	0.85
SUP T2	2.86	3.09	0.62	−1.02	0.87
SUP T3	2.89	3.14	0.59	−1.11	0.88
SPIR T1	5.28	4.04	0.06	−1.30	0.85
SPIR T2	5.29	4.11	0.05	−1.36	0.86
SPIR T3	5.14	4.20	0.12	−1.37	0.88
ELF T1	4.05	2.67	0.16	−0.91	0.60
ELF T2	4.03	2.71	0.16	−0.98	0.60
ELF T3	3.94	2.80	0.23	−1.01	0.65
PRE T1	4.93	3.75	0.19	−1.08	0.83
PRE T2	4.86	3.77	0.24	−1.09	0.83
PRE T3	4.71	3.84	0.27	−1.11	0.85

PB, Paranormal Belief; PSS, Perceived Stress; SC, Somatic Complaints; TRB, Traditional Religious Belief; WITCH, Witchcraft; SUP, Superstition; SPIR, Spirituality; ELF, Extraordinary Lifeforms; PRE, Precognition.

#### Perceived Stress Scale

The Perceived Stress Scale (PSS-10; Cohen and Williamson, 1988) is a 10-item measure of individual perceptions of uncontrollability and unpredictability (life pressure) over the past month. Within the PSS-10 items appear as questions (e.g. ‘how often have you felt that you were on top of things?’), and respondents indicate their level of agreement by completing a 5-point Likert Scale (i.e. 0 = Never and 4 = Very Often). Two subscales frequently emerge from analyses of factor structure: PSS Distress (indexing feelings of distress) and PSS Coping (indexing ability to cope with problems) (Andreou et al., 2011). The PSS-10 has established psychometric properties (i.e. reliability and validity) (Denovan et al., 2019). After the computation of a total raw PSS-10 score, PSS Coping items were reverse-scored according to the scale author instructions (Cohen and Williamson, 1988). See Table 1 for internal reliability information.

#### Somatic Symptom Scale-8

The Somatic Symptom Scale-8 (SSS-8; Gierk et al., 2014) is an 8-item instrument that evaluates the presence and severity of somatic symptoms during the past 7-days. Problems appear in the form of symptoms (e.g. ‘Dizziness’) and respondents indicate occurrence/intensity on a 5-point Likert scale (i.e. 0 = Not at all and 4 = Very Much). The SSS-8 is an established, widely used, psychometrically robust measure (see Table 1 for study internal reliability) (Gierk et al., 2014).

#### Procedure

Respondents clicked a web link that directed them to the study information. This outlined the general purpose of the research project and the requirement to complete measures on three separate time points 2 months apart. Accordingly, respondents who agreed to participate provided an identification number, which allowed response matching across trials. Following the collation of the data sheet, the identification number was deleted. For respondents who progressed to the online measures, the procedure was the same at each time point. To reduce potential methodological issues, the investigators implemented procedural remedies. Firstly, to lessen potential common method variance, psychological distance was created between scales by accentuating construct uniqueness. Secondly, to reduce social desirability and evaluation apprehension, instructions stated that there were no correct or preferred responses. Finally, rotation of sections across respondents controlled for order effects. The demographic section was always completed first. Additionally, respondents were directed to take their time, complete measures at their own pace, carefully read items and complete all questions.

### Ethics statement

The Faculty of Health, Psychology and Social Care Ethics Committee at Manchester Metropolitan University granted ethical approval (Project ID, 25390).

### Analysis

Data screening examined normality. Then, internal reliability of the RPBS was evaluated prior to examining structural validity of the study scales (RPBS, PSS-10 and SSS-8) using confirmatory factor analysis (CFA). Subsequently, longitudinal invariance tests were performed to assess the notion that properties of the study scales did not significantly differ over time. Latent construct scores derived from the item-level analyses were implemented in a test of temporal (test–retest) reliability and predictive validity using path analysis. Path analysis employed a statistical model assessing the quantity of variance in perceived stress (PSS) and somatic complaints (SC) explained/predicted by paranormal belief (PB, as measured by the RPBS) over time.

In addition to inspection of explained variance, analysis evaluated the quality of model fit. This included consideration of a range of indices, specifically Confirmatory Fit Index (CFI), Standardized Root-Mean-Square Residual (SRMR) and Root-Mean-Squared Error of Approximation (RMSEA). Satisfactory thresholds for these are CFI > 0.90, SRMR <0.08 and RMSEA <0.06 (Hu and Bentler, 1999). These criteria were additionally employed when examining structural validity and invariance. For invariance, CFI differences ≤0.01 alongside RMSEA changes ≤0.015 are satisfactory (Chen, 2007).

## Results

### Data screening and internal reliability

Assessment of normality (Table 1) revealed that none of the study variables possessed skewness or kurtosis greater than the recommended threshold of +2 or-2 (Field and Miles, 2010). However, Mardia’s (1970) multivariate kurtosis was 63.51, suggesting non-normality. Accordingly, analysis employed the bootstrap method (with 1,000 resamples) to compute reliable standard errors and estimates (Byrne, 2010). Internal reliability of the RPBS was high at each time point (Time 1 *α* = 0.94, Time 2 *α* = 0.94, Time 3 *α* = 0.95). Internal reliability of the seven RPBS factors was satisfactory in all instances apart from Extraordinary Lifeforms. Concerns regarding this subscale in terms of its appropriateness and reliability have been raised previously (Drinkwater et al., 2017). The remaining factors demonstrated satisfactory reliability.

### Structural validity

Prior to assessing latent relationships among the study constructs, structural validity involved examination of the RPBS (correlated seven-factor, higher-order seven-factor and a one-factor model as a null test), the SSS-8 (one-factor model) and the PSS-10 (correlated two-factor and a one-factor model as a null test). These conceptualisations of the study measures exist in the research literature (e.g. Tobacyk, 2004; Andreou et al., 2011; Denovan et al., 2018).

The correlated seven-factor RPBS solution demonstrated acceptable fit, χ^2^ (278, *N* = 1,665) = 2349.98, *p* < 0.001, CFI = 0.92, SRMR = 0.04, RMSEA = 0.06 (95% CI of 0.06 to 0.07). Similarly, the higher-order seven-factor model revealed satisfactory fit, χ^2^ (292, *N* = 1,665) = 2307.78, *p* < 0.001, CFI = 0.92, SRMR = 0.04, RMSEA = 0.06 (95% CI of 0.06 to 0.07). Fit was similar to the correlated solution. However, Akaike’s Information Criterion (a model comparison index) was marginally lower for the higher-order model (2505.78 vs. 2519.98), suggesting a more parsimonious solution. The one-factor model was unsatisfactory on all indices but SRMR, χ^2^ (299, *N* = 1,665) = 4639.75, *p* < 0.001, CFI = 0.83, SRMR = 0.05, RMSEA = 0.09 (95% CI of 0.09 to 0.10).

The two-factor correlated PSS-10 model evidenced good fit, χ^2^ (34, *N* = 1,665) = 294.23, *p* < 0.001, CFI = 0.97, SRMR = 0.03, RMSEA = 0.06 (95% CI of 0.06 to 0.07). However, a one-factor conceptualisation was unsatisfactory, χ^2^ (35, *N* = 1,665) = 2608.32, *p* < 0.001, CFI = 0.73, SRMR = 0.16, RMSEA = 0.21 (95% CI of 0.20 to 0.21). Conversely, a one-factor solution was suitable for the SSS-8 on all indices but RMSEA, χ^2^ (20, *N* = 1,665) = 457.84, *p* < 0.001, CFI = 0.92, SRMR = 0.04, RMSEA = 0.10 (95% CI of.10 to 0.12).

### Longitudinal invariance

The first stage of longitudinal invariance testing involved assessing the baseline (configural) model for each construct. The RPBS demonstrated satisfactory invariance of form (Table 2). Progression from the test of form to factor loadings (metric) revealed no change in CFI, and a change of 0.001 in RMSEA. Constraining the intercepts (scalar level) additionally evidenced no CFI change alongside a change of 0.001 in RMSEA. Lastly, constraining residual variance to equality revealed a 0.002 CFI change and a 0.001 RMSEA change.

**Table 2:** Fit indices for longitudinal invariance models.

Model	*χ* ^2^	Δ*χ*^2^	*df*	Δ*df*	CFI	ΔCFI	SRMR	RMSEA (90% CI)	ΔRMSEA
**RPBS**									
Configural	7793.71**	–	876	–	0.92	–	0.04	0.04 (0.03–0.04)	–
Metric	7827.56**	33.85	914	38	0.92	None	0.04	0.04 (0.03–0.04)	0.001
Scalar	7890.25**	62.69	966	52	0.92	None	0.04	0.04 (0.03–0.04)	0.001
Residual	8130.05**	239.79*	1,046	80	0.92	0.002	0.04	0.04 (0.03–0.04)	0.001
**PSS-10**									
Configural	968.54**	–	102	–	0.97	–	0.03	04 (0.03–0.04)	–
Metric	983.68**	15.13	118	16	0.97	None	0.03	03 (0.03–0.04)	0.003
Scalar	1014.94**	31.25	138	20	0.97	None	0.03	03 (0.03–0.04)	0.002
Residual	1061.55**	77.87	164	46	0.97	0.001	0.04	03 (0.03–0.04)	0.003
**SSS-8**									
Configural	647.49**	–	54	–	0.97	–	0.04	0.04 (0.04–0.05)	–
Metric	662.77**	15.27	68	14	0.97	0.001	0.04	0.04 (0.03–0.04)	0.005
Scalar	686.08**	23.31	84	16	0.97	None	0.04	0.03 (0.03–0.04)	0.004
Residual	751.05**	64.96	106	22	0.96	0.002	0.03	0.03 (0.03–0.04)	0.003

**χ^2^ significant at *p* < 0.001, *Δχ^2^ significant at *p* < 0.001.

For the PSS-10, satisfactory configural invariance across time existed. No meaningful CFI difference existed at the metric level (factor loadings) with a 0.003 RMSEA difference. Similarly, the scalar level exhibited no CFI change and a 0.002 RMSEA difference. At the residual level, a 0.001 CFI change and a 0.003 RMSEA change occurred. The SSS-8 exhibited satisfactory fit at the configural level. Progression to the metric level revealed a 0.001 CFI difference and 0.005 RMSEA difference. The scalar model exhibited no CFI change and a 0.004 RMSEA difference. Lastly, the test of equal residuals revealed a 0.002 CFI difference and a 0.003 RMSEA change. These results indicated satisfactory invariance across the time intervals in this study for the RPBS, PSS-10 and SSS-8.

### Test–retest reliability

The test–retest reliability coefficients (Pearson and Spearman’s Rho) were computed in relation to the latent construct scores derived from analysis of the statistical models. In contrast to total questionnaire scores, these are uncontaminated by measurement error (Lodder et al., 2022). Across the three time points for the RPBS (assessing paranormal belief, PB), test–retest was in the moderate to high range (> 0.70). Specifically, Time 1 and Time 2 *r* = 0.77, *r_s_* = 0.78, Time 1 and Time 3 *r* = 0.79, *r_s_* = 0.79, Time 2 and Time 3 *r* = 0.83, *r_s_* = 0.84. Additional scale and test–retest reliability coefficients appear in Table 3. Test–retest reliability was moderate to high across factors.

**Table 3:** Test–retest for RPBS, PSS, PSS factors, SSS-8 and RPBS factors using latent construct scores.

Variable	Test–retest T1–T2 *r* (*r_s_*)	Test–retest T1–T3 *r* (*r_s_*)	Test–retest T2–T3 *r* (*r_s_*)
PB	0.77** (0.78**)	0.79** (0.79**)	0.83** (0.84**)
PSS Distress	0.79** (0.79**)	0.77** (0.79**)	0.80** (0.81**)
PSS Coping	0.60** (0.61**)	0.60** (0.60**)	0.64** (0.64**)
SC	0.79** (0.81**)	0.78** (0.79**)	0.80** (0.81**)
TRB	0.76** (0.78**)	0.78** (0.79**)	0.82** (0.83**)
PSI	0.74** (0.75**)	0.76** (0.76**)	0.80** (0.81**)
WITCH	0.75** (0.75**)	0.77** (0.77)**	0.82** (0.82**)
SUP	0.75** (0.77**)	0.74** (0.77**)	0.80** (0.83**)
SPIR	0.77** (0.77**)	0.78** (0.78**)	0.82** (0.83**)
ELF	0.77** (0.78**)	0.79** (0.79**)	0.83** (0.84**)
PRE	0.77** (0.78**)	0.79** (0.79**)	0.83** (0.84**)

PB, Paranormal Belief; PSS, Perceived Stress; SC, Somatic Complaints; TRB, Traditional Religious Belief; WITCH, Witchcraft; SUP, Superstition; SPIR, Spirituality; ELF, Extraordinary Lifeforms; PRE, Precognition. **p < 0.001.

#### Internal construct validity

Scrutiny of associations at each time point using latent scores revealed small to moderate significant positive correlations between RPBS and PSS Distress (Time 1 = 0.20, Time 2 = 0.19, Time 3 = 0.19), and RPBS and SSS-8 (Time 1 = 0.19, Time 2 = 0.20, Time 3 = 0.20). Small significant negative correlations existed between RPBS and PSS Coping (Time 1 = −0.15, Time 2 = −0.19, Time 3 = −0.18).

### Predictive validity

The fit of the PB - PSS and SC predictive model over time, using the latent factor scores, was satisfactory on all indices apart from RMSEA, χ^2^ (28, *N* = 1,665) = 1095.67, *p* < 0.001, CFI = 0.93, SRMR = 0.05, RMSEA = 0.15 (95% CI of 0.14 to 0.15). Inspection of modification indices revealed that a superior data-fit (and satisfactory RMSEA) occurred if error terms between Time 2 and Time 3 were allowed to correlate. However, this change conflicted with the depiction of temporal order within the analysis, and subsequently was not permitted.

**Figure 1:**
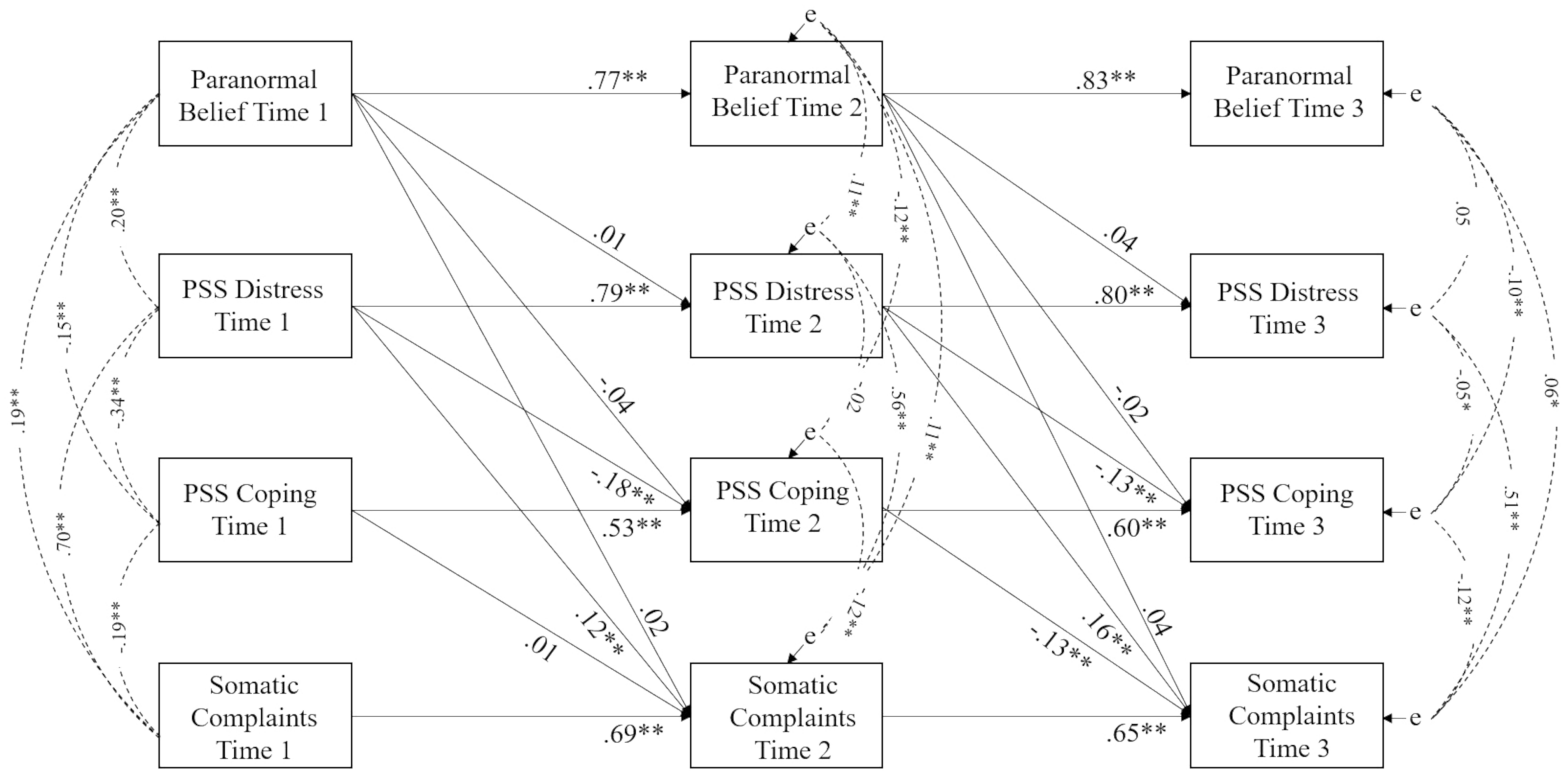
Predictive model of paranormal belief over time in relation to well-being (perceived stress and somatic complaints) using latent construct scores. *Note.* Standardised regression weights between variables are shown. Error is indicated by ‘e’. * *p* < 0.05, ** *p* < 0.001 using Bootstrapping significance estimates (1,000 resamples).

Associations between variables at each time point (Figure 1) were significant and positive in all instances but for Time 2 PSS Distress and PSS Coping (*r* of −0.02), and Time 3 PB and PSS Distress (*r* of 0.05). In addition, paths between Time 1, Time 2 and Time 3 were significant for each respective construct (e.g. PB Time 1, Time 2, Time 3, etc.). Explained variance ranged from 38 to 69%. However, predictive relationships over time from PB to SC, PSS Distress and PSS Coping were not significant. Predictive paths were significant from PSS Distress to SC over time.

Accordingly, a model was specified in which paths from PSS to SC and PSS and SC sequential paths (e.g. PSS Time 1 to PSS Time 2, etc.) were fixed to zero to assess the quantity of explained variance in PSS Distress, PSS Coping and SC due to PB. This was low: 2.6 and 3.5% in PSS Distress at Time 2 and Time 3, 2.5 and 2.4% in PSS Coping at Time 2 and Time 3 and 3 and 4.2% in SC at Time 2 and Time 3. This indicated that a small proportion of variance in the outcomes over time was attributable to PB.

## Discussion

The RPBS at both global and subscale levels demonstrated acceptable test–retest reliability (*r* > 0.70) (Del Rosario and White, 2005). This indicated that the RPBS possesses adequate temporal consistency and that belief in the paranormal remained stable across an extended time interval (6 months). Although the subscale correlations observed in the present study were lower than those reported by Tobacyk (2004), these conclusions are generally commensurate with the findings of the RPBS validation study.

The lower correlations within the present sample are attributable to the large heterogeneous sample used. In comparison, Tobacyk (2004) assessed test–retest over a 4-week period using a small, homogeneous sample of students. Despite this, variations in correlation size across intervals in this study remained within the acceptable range. Explicitly, Precognition 77 to 0.83, Witchcraft 0.75 to 0.82, Extraordinary Lifeforms 0.77 to 0.83, Traditional Religious Belief 0.76 to 82, Psi 0.74 to 0.80, Superstition 0.74 to 0.80 and Spiritualism 0.77 to 0.82. The overall scale test–retest was 0.77 to 0.83. Moreover, a longer period was employed in this study between each successive interval (2 months), and adequate stability coefficients additionally existed at 4 months (i.e. between Time 1 and 2).

In addition, longitudinal invariance tests revealed that the RPBS measurement properties did not significantly alter over time. This has not previously been established for the RPBS, and it indicated that any changes at the construct level were not contaminated by measurement bias (Lodder et al., 2022). Furthermore, a more accurate estimate of temporal stability existed in comparison with preceding research (e.g. Tobacyk, 2004) utilising sum scores (which risk contamination by measurement error), limiting the likelihood of under or overestimating stability over time.

Evidence of satisfactory stability overall supports the assumption that paranormal belief would remain relatively stable over time. Furthermore, a greater stability coefficient existed for subfactors including Traditional Religious Belief, with lower coefficients occurring for subfactors indexing superstition and magical thinking. This is consistent with the view that religious beliefs are typically enduring, whereas superstition and magical thinking are more likely to vary in accordance with situational factors (Padgett and Jorgenson, 1982; Dagnall et al., 2007).

Paranormal belief was similarly associated with outcome measures (PSS Distress, PSS Coping and Somatic Complaints) at each of the three assessments points, suggesting that the relationships were stable. Although most correlations were significant, they were small (*r* = −0.15 to.20, Gignac and Szodorai, 2016). Paranormal belief furthermore explained only 2.6 to 3.5% PSS Distress variance, 2.4 to 2.5% PSS Coping variance and 3 to 4.2% of the variance in Somatic Complaints over time. Essentially, the effects of paranormal belief on perceived stress and somatic complaints were weak in isolation, which supports the need to include additional variables to comprehend how this influences allied health outcomes/well-being. Indeed, these findings were consistent with recent work that has found that paranormal belief, in the context of negative well-being is typically benign (Dagnall et al., 2022a,b,c,d). Moreover, this supports the notion that adverse outcomes allied to paranormal belief arise from interactions with productive and disorganised cognitive-perceptual and psychopathological factors.

### Limitations

Primary limitations of the study include the composition of the sample and the utilised measures. With regard to the sample, because this was sourced from a willing pool of respondents, there is the potential for self-selecting bias. Although this approach made pragmatic sense, a more naturally occurring sample would have produced findings that were increasingly representative of the population of interest. Relatedly, insufficient sample demographics were collected in relation to the study variables. Information including ethnicity, religious inclination and physical health would have been important to appreciate relative to paranormal belief and perceived stress and somatic complaints. As a minimum, this information should be controlled for in future research.

A second issue relates to the timing of the research. Data were collected in 2020/2021 surrounding the COVID-19 pandemic, and research indicates that increased levels of distress and stress existed during this period (e.g. Charles et al., 2021). Therefore, this should be appreciated in light of the findings. However, it is important to note that the sample reported perceived stress levels that were comparable to previously established norms with a general population sample (*M* = 14.20, *SD* = 6.20; Cohen, 1994).

In terms of the measures, the present study focused only on Perceived Stress and Somatic Complaints. Although robust scales were used, these represent a narrow conceptualisation of health. It would be important for future research to incorporate additional health-related features, such as the construct of psychological well-being. This could use the Psychological Well-Being Scale (Ryff and Keyes, 1995), which includes several domains including self-acceptance, purpose in life, autonomy and mastery. Moreover, recent research has established a link between paranormal belief and meaning in life (FioRito et al., 2021).

## Data availability statement

The raw data supporting the conclusions of this article will be made available by the authors, without undue reservation.

## Ethics statement

The studies involving human participants were reviewed and approved by the Manchester Metropolitan University Faculty of Health, Psychology and Social Care Ethics Committee (December 2020; Project ID 25390). Written informed consent for participation was not required for this study in accordance with the national legislation and the institutional requirements. Written informed consent was obtained from the individual (s) for the publication of any potentially identifiable images or data included in this article. The patients/participants provided their written informed consent to participate in this study.

## Author contributions

AD and ND designed the study. ND provided conceptual input, developed the theoretical context, summarised findings, and edited all sections. AD collated measurement scales, arranged data collection, and performed analyses. KD and AD revised the final manuscript and prepared the draft submission. All authors contributed fully to the article and approved the final version.

## Funding

We would like to thank the BIAL Foundation for their support of this project (grant number: 123/20).

## Conflict of interest

The authors declare that the research was conducted in the absence of any commercial or financial relationships that could be construed as a potential conflict of interest.

## Publisher’s note

All claims expressed in this article are solely those of the authors and do not necessarily represent those of their affiliated organizations, or those of the publisher, the editors and the reviewers. Any product that may be evaluated in this article, or claim that may be made by its manufacturer, is not guaranteed or endorsed by the publisher.
